# SLEEP DISTURBANCES AMONG UNIVERSITY STUDENTS : A TUNISIAN STUDY

**DOI:** 10.1192/j.eurpsy.2023.2339

**Published:** 2023-07-19

**Authors:** M. Turki, J. Firas, H. E. Mhiri, F. Sahnoun, A. Guermazi, S. Ellouze, N. Halouani, J. Aloulou

**Affiliations:** 1 Psychiatry “B” department, Hedi Chaker university hospital, Sfax, Tunisia

## Abstract

**Introduction:**

Poor sleep quality is a major health problem worldwide. University students tend to suffer from problems of sleep regularity, quantity and quality, which can affect their academic performance, and have a serious impact on their psychological and physical well-being.

**Objectives:**

The aim of this study was to assess the prevalence of insomnia among Tunisian university students, and to identify its associated factors.

**Methods:**

We conducted a cross-sectional web-based study among university students from several Tunisian faculties. Data were collected using a questionnaire spread throughout social media (Facebook), using the Google Forms® platform, during September and October 2022.

We used the “Insomnia Severity Index” (ISI) to assess the severity of insomnia.

**Results:**

A total of 144 students completed the questionnaire. Their mean age was 23.38±3.27 years, with a sex-ration (F/M) of 2.8. Among them, 70.1% were single and 68.8% lived with family. Among our participants, 10.4% were followed for chronic somatic disease, 11.1% for chronic mental disease, while 29.2% have already been diagnosed and treated for sleep disturbances.

ISI showed that 72.2% of students suffered from insomnia: 45.1% Subthreshold insomnia, 19.4% moderate clinical insomnia and 7.6% severe clinical insomnia. Insomnia was significantly more frequent among psychoactive substances users (75.7% vs 57.6%; p=0.043). ISI scores were significantly higher among cannabis users (17.4 vs 11.06; p=0.025).

**Conclusions:**

Our study highlighted that insomnia is prevalent within the university student population, and psychoactive substances consumption seems to worsen it. Thus, when designing interventions to improve sleep quality among students, the main determinants need to be taken into consideration.

**Disclosure of Interest:**

None Declared

